# Endometriosis and Oocyte Quality: Morphological Alterations, Developmental Competence, and Modifiable Strategies for Reproductive Longevity

**DOI:** 10.3390/cells15030296

**Published:** 2026-02-04

**Authors:** Martina Contestabile, Ilaria Marzi, Calogero Mangione, Ferdinando Franzoni, Paolo Giovanni Artini, Simona Daniele

**Affiliations:** 1 Department of Pharmacy, University of Pisa, 56126 Pisa, Italy; m.contestabile@student.unisi.it (M.C.); c.mangione4@studenti.unipi.it (C.M.); simona.daniele@unipi.it (S.D.); 2 Division of Gynecology and Obstetrics, Department of Clinical and Experimental Medicine, University of Pisa, 56126 Pisa, Italy; ilaria.marzi@ao-pisa.toscana.it; 3 Division of Sports Medicine, Department of Clinical and Experimental Medicine, University of Pisa, 56126 Pisa, Italy; ferdinando.franzoni@unipi.it

**Keywords:** endometriosis, oocyte competence, mitochondria, follicular microenvironment, oxidative stress, epigenetic regulation, fertility preservation

## Abstract

Endometriosis is a chronic, estrogen-dependent inflammatory disorder that is increasingly recognized as a systemic condition with profound implications for female reproductive potential. In addition to pelvic distortion and impaired folliculogenesis, growing evidence indicates that intrinsic alterations in oocyte morphology, mitochondrial function, and developmental competence contribute to infertility. The disease is driven by a multifactorial interplay of somatic mutations, epigenetic remodeling, immune dysregulation, and aberrant steroid signaling, which together create a pro-inflammatory, oxidative, and fibrotic microenvironment. Elevated cytokines, reactive oxygen species, and disrupted granulosa-cell function within the follicular niche impair meiotic progression, cytoplasmic maturation, and mitochondrial integrity, potentially accelerating oocyte aging and diminishing reproductive longevity. Epigenetic and post-transcriptional disturbances—including altered DNA methylation, histone modifications, and RNA-splicing defects—further reinforce estrogen dominance, progesterone resistance, and impaired decidualization, with downstream consequences for ovarian–endometrial communication. Although morphological abnormalities have been documented in oocytes from women with endometriosis, clinical outcomes remain heterogeneous, highlighting the need for integrative models that connect molecular alterations to functional reproductive endpoints. A deeper understanding of these mechanisms is essential for identifying biomarkers of oocyte competence and modifiable strategies—ranging from nutritional optimization to reduction of environmental risk factors—in clinical care to safeguard the reproductive potential of women with endometriosis.

## 1. Introduction

Endometriosis is a chronic, estrogen-dependent, inflammatory, and systemic gynecological disease characterized by the presence of endometrial-like tissue outside the uterine cavity. It affects more than 10% of women of reproductive age and represents one of the most common disorders associated with infertility and chronic pelvic pain [1,2]. Despite substantial advances in diagnostic and therapeutic approaches, the mechanisms through which endometriosis impairs reproductive competence remain only partially understood. Increasing evidence suggests that endometriosis should not be viewed merely as an ectopic endometrial disorder, but rather as a condition driven by the clonal expansion of genetically and/or epigenetically altered cells exhibiting aberrant aromatase activity and progesterone resistance [2]. This conceptual shift supports a systemic and multifactorial understanding of the disease.

Infertility associated with endometriosis arises from multiple interacting mechanisms, whose relative contribution varies according to disease phenotype, stage, lesion localization, ovarian involvement, and the extent of pelvic distortion [3]. Traditional explanations include pelvic anatomical distortion, impaired folliculogenesis, immune dysregulation, and oxidative stress. However, accumulating evidence indicates that intrinsic alterations in oocyte quality and developmental competence may also play a role, potentially originating from the inflammatory, angiogenic, and fibrotic microenvironment characteristic of endometriotic lesions [4].

A major clinical controversy concerns whether endometriosis affects oocyte morphology. Several studies have reported a higher frequency of abnormal zona pellucida structure, cytoplasmic granularity, and vacuolization in oocytes retrieved from affected women, suggesting disturbances in meiotic progression and cytoplasmic maturation [5]. These features have been proposed as potential indicators of reduced oocyte competence. Conversely, other investigations have shown comparable fertilization rates, blastocyst development, euploidy, implantation, and live birth outcomes between women with and without endometriosis, challenging the assumption that morphological abnormalities necessarily translate into impaired reproductive potential [6]. This discrepancy highlights the complexity of establishing reliable morphology-based predictors of oocyte quality in this population.

Emerging molecular evidence further suggests that endometriosis is sustained by profound epigenetic dysregulation, involving alterations in DNA methylation, histone modifications, and non-coding RNA networks. These changes contribute to the inflammatory, estrogen-dependent, and progesterone-resistant phenotype of the disease and may influence both ovarian function and oocyte competence [7]. Collectively, these observations indicate that endometriosis-related infertility does not stem from a single pathogenic mechanism but rather from the dynamic interplay among inflammation, epigenetic remodeling, impaired ovarian reserve, and microenvironment-mediated effects on the oocyte. A deeper understanding of these interactions is essential for refining prognostic models and developing personalized reproductive strategies.

### 1.1. Molecular Alterations in Endometriosis

Contemporary models propose that endometriosis arises from interconnected genomic, epigenetic, and environmental influences, rather than from a single causative event. In this framework, normal endometrial epithelial cells frequently have somatic mutations that confer selective advantages, enabling survival during retrograde menstruation and adaptation to the hostile peritoneal environment. Only a subset of genetically or epigenetically advantaged cells is able to evade anoikis and establish ectopic lesions, whereas the majority of refluxed cells undergo apoptosis. This process reflects a Darwinian paradigm of mutation, selection, and adaptation [8]. Importantly, clonal selection and molecular adaptation likely precede clinical diagnosis by several years, suggesting that genomic and epigenetic alterations may already affect endometrial receptivity and ovarian–endometrial communication during early reproductive life, well before overt disease becomes apparent [2,8].

Comprehensive multi-omics analyses have revealed overlapping molecular signatures between endometriosis and reproductive failure, including recurrent spontaneous abortion (RSA) and recurrent implantation failure (RIF). Hub genes such as FXYD1, along with genes implicated in endothelial–mesenchymal transition (EMT) including ITGB1, VIM, and FGF2, have been consistently identified across independent transcriptomic datasets [9,10,11]. These findings highlight convergent dysregulation of pathways involved in extracellular matrix (ECM) remodeling, angiogenesis, immune regulation, steroid hormone signaling, and metabolic adaptation [12].

Somatic mutations and clonal expansion confer a proliferative and survival advantage to ectopic endometrial cells. Cancer-like molecular features, including alterations in ARID1A, PIK3CA, and PTEN, and dysregulation of the PI3K–AKT, MAPK, and NF-κB pathways, overlap with mechanisms implicated in impaired endometrial receptivity, defective decidualization, and progesterone resistance [13]. These observations support a biological continuum linking benign endometriotic disease, reproductive failure, and, in selected contexts, malignant transformation.

Although these alterations are primarily described in eutopic and ectopic endometrial tissue, increasing evidence suggests that similar molecular disturbances may extend to the ovarian microenvironment, potentially affecting follicular development, oocyte competence, and local inflammatory signaling [4,10].

In this context, somatic mutations and genetic susceptibility establish a permissive framework that is subsequently amplified and stabilized by epigenetic remodeling, generating self-reinforcing transcriptional programs that sustain lesion survival and interfere with reproductive processes [8,10,13].

**Figure 1:**
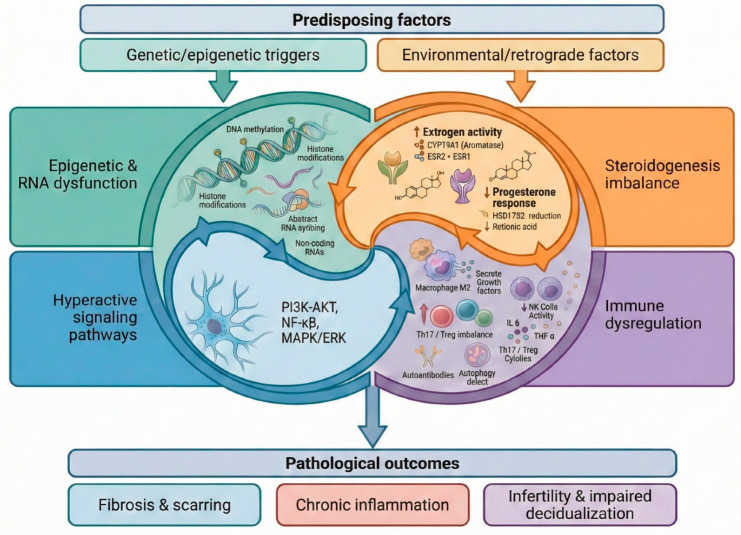
**Integrated molecular mechanisms underlying endometriosis and associated reproductive dysfunction**. Schematic overview of the interconnected genetic, epigenetic, hormonal, immune, and signaling mechanisms involved in endometriosis. Epigenetic and RNA splicing alterations, steroidogenic imbalance, immune dysregulation, and hyperactivation of PI3K–AKT, MAPK/ERK, and NF-κB pathways interact to sustain chronic inflammation, fibrosis, progesterone resistance, and impaired decidualization, ultimately contributing to infertility. Adapted from [8,11,12].

The genetic, epigenetic, immune, and signaling alterations underlying endometriosis act in a highly interconnected manner, as schematically summarized in Figure 1.

### 1.2. Epigenetic and RNA Splicing

Beyond genetic susceptibility and somatic mutations, epigenetic remodeling represents a central mechanism driving the establishment, persistence, and reproductive consequences of endometriosis [14]. Epigenetic alterations, including aberrant DNA methylation, histone modifications, and dysregulated non-coding RNA networks, profoundly reshape the transcriptional landscape of endometriotic stromal and epithelial cells, generating stable disease-specific gene expression programs [15].

Aberrant DNA methylation of promoter regions in endometriotic stromal cells has been linked to dysregulated expression of key genes, including Estrogen receptor alpha (ERα), Estrogen receptor beta (ERβ), Progesterone Receptor (PGR), Homeobox A10 (HOXA10), and Cytochrome P450 Family 19 Subfamily A Member 1 (CYP19A1), contributing to estrogen dominance, progesterone resistance, and aberrant inflammatory signaling [8,15]. Specifically, CpG islands in the ERβ promoter are hypomethylated, whereas those in ERα and PGR promoters are hypermethylated compared with eutopic endometrium, reinforcing the characteristic hormonal imbalance of the disease [8]. These epigenetic alterations impair progesterone-mediated decidualization, disrupt endometrial receptivity, and establish a direct mechanistic link between lesion biology and reproductive dysfunction [8]. Histone modifications further cooperate with DNA methylation to reinforce inflammatory and pro-survival signaling pathways, including Nuclear Factor kappa-light-chain-enhancer of activated B cells (NF-κB), Transforming Growth Factor beta TGF-β, and Phosphoinositide 3-kinase–Protein Kinase B (PI3K–AKT) and Protein Kinase B (AKT) cascades. Together, these epigenetic alterations create a permissive chromatin environment that sustains lesion survival, immune evasion, and fibrotic remodeling, while limiting the capacity of the endometrium to undergo appropriate cyclic differentiation [16].

In addition to transcriptional control, post-transcriptional regulation has emerged as a critical layer of molecular dysregulation in endometriosis. Recent transcriptomic analyses have identified widespread alterations in RNA splicing programs within endometriotic tissue, implicating spliceosome components and splicing regulators in disease pathogenesis. Dysregulated alternative splicing affects genes involved in hormone responsiveness, cell-cycle control, inflammatory signaling, and extracellular matrix organization, potentially generating protein isoforms with altered or dominant-negative functions [17,18]. Notably, aberrant splicing produces protein isoforms with altered or dominant-negative functions, potentially amplifying pathogenic signaling cascades such as TGF-β/Smad, PI3K–AKT, and NF-κB [19,20,21]. These isoform shifts may further reinforce progesterone resistance, fibrotic remodeling, and impaired decidualization, linking molecular alterations directly to reproductive dysfunction. Importantly, epigenetic and splicing alterations appear to be tightly interconnected. DNA methylation and chromatin structure influence splice-site selection, while aberrant splicing can, in turn, modulate epigenetic regulators, establishing a reciprocal regulatory loop that amplifies molecular instability and contributes to cellular heterogeneity and disease persistence [17,22]. Complementary epigenome-wide association studies and systematic reviews further demonstrate aberrant methylation of genes involved in immune regulation, steroid signaling, angiogenesis, and extracellular matrix remodeling. Collectively, these findings support a model in which epigenetic and post-transcriptional dysregulation not only sustains lesion persistence but may also influence ovarian–endometrial crosstalk, the integrity of the follicular microenvironment, and oocyte developmental competence [20,23].

Importantly, epigenetic modifications and alternative RNA splicing exert direct effects on the ovarian microenvironment and oocyte quality. Aberrant DNA methylation and histone modifications in granulosa and cumulus cells alter the transcription of key regulators of steroid hormone signaling (ERα, ERβ, PGR, and HOXA10) and cell-cycle progression (CCNB1, CDC25C, and AURKA), thereby impairing meiotic spindle assembly, cytoplasmic maturation, and chromosome segregation [8,15]. Dysregulated splicing generates protein isoforms with altered or dominant-negative functions, amplifying pathogenic signaling through TGF-β/Smad, PI3K–AKT, and NF-κB pathways. These alterations compromise mitochondrial function and ATP production, reduce oocyte metabolic competence, and increase susceptibility to oxidative stress [17,18,19,20].

Moreover, epigenetic silencing of ERα and PGR reinforces aberrant splicing and promotes local estrogen dominance and progesterone resistance, establishing a self-perpetuating pathogenic loop. These molecular disturbances also elevate local pro-inflammatory cytokines (IL-6, TNF-α) within the follicular microenvironment, further disrupting cumulus–oocyte communication and meiotic progression. Multi-omic analyses of cumulus–oocyte complexes from women with endometriosis confirm that these epigenetic and splicing abnormalities are associated with reduced oocyte developmental potential, lower fertilization rates, and impaired embryo quality. Altogether, these findings delineate a mechanistic pathway linking epigenetic and post-transcriptional dysregulation directly to impaired oocyte competence and adverse reproductive outcomes in endometriosis [22,23].

### 1.3. Immune Dysregulation in Endometriosis

Endometriosis is increasingly recognized as a chronic immune-mediated inflammatory disease, characterized by profound alterations in both innate and adaptive immune responses that contribute to lesion establishment, persistence, and reproductive dysfunction [24,25]. Rather than representing a state of generalized immune activation, endometriosis is marked by a paradoxical immune phenotype in which chronic inflammation coexists with impaired immune surveillance, allowing ectopic endometrial cells to evade clearance.

Innate immune cells play a central role in the pathogenesis of endometriosis. Peritoneal macrophages are increased in number and exhibit a skewed polarization toward an M2-like, pro-angiogenic and pro-fibrotic phenotype. These macrophages secrete elevated levels of cytokines and growth factors, including Interleukins (IL)-6, IL-8, TNF-α, TGF-β, VEGF, and prostaglandins, which promote lesion survival, angiogenesis, fibrosis, and nerve sensitization [24,25,26]. At the same time, macrophage phagocytic capacity is impaired, limiting the clearance of refluxed endometrial debris.

Natural killer (NK) cell dysfunction represents another hallmark of endometriosis-associated immune escape. Multiple studies have demonstrated reduced NK cell cytotoxicity in the peritoneal fluid and eutopic endometrium of affected women, driven by altered expression of activating and inhibitory receptors and by suppressive cytokines within the lesion microenvironment [24,25,26]. This defect allows ectopic endometrial cells to evade immune-mediated apoptosis during retrograde menstruation.

Neutrophils and mast cells are also enriched in endometriotic lesions and contribute to sustained inflammation through the release of reactive oxygen species (ROS), proteases, and pro-inflammatory mediators. Mast cell–nerve fiber interactions further link immune activation to pain generation and neuroinflammation [25].

Adaptive immune responses are similarly dysregulated in endometriosis. T cell populations show an imbalance characterized by increased Th1 and Th17 responses, reduced regulatory T cell (Treg) function, and altered cytokine profiles that favor chronic inflammation and tissue remodeling [25,26]. Elevated levels of IL-17 and IFN-γ have been implicated in angiogenesis, fibrosis, and resistance to apoptosis within lesions.

B cell activation and autoantibody production further support the concept of endometriosis as an autoimmune-like condition. Women with endometriosis display an increased prevalence of circulating autoantibodies and higher comorbidity with autoimmune diseases, including systemic lupus erythematosus, rheumatoid arthritis, and autoimmune thyroid disorders [27]. These observations reinforce the view that loss of immune tolerance contributes to disease persistence and systemic manifestations.

Immune dysregulation in endometriosis is tightly intertwined with hormonal signaling. Estrogen enhances inflammatory responses by activating macrophages, promoting Th17 differentiation, and suppressing NK cell cytotoxicity, thereby reinforcing immune tolerance toward ectopic lesions. In contrast, progesterone normally exerts anti-inflammatory and immunomodulatory effects; however, progesterone resistance in endometriosis disrupts this regulatory balance [28].

Recent work has highlighted the ovarian hormones–autophagy–immunity axis as a critical regulator of endometrial homeostasis and lesion survival. Altered autophagic flux in endometriotic cells influences antigen presentation, cytokine secretion, and immune cell recruitment, further contributing to immune escape and chronic inflammation [28].

Immune abnormalities associated with endometriosis extend beyond lesion biology and directly impact reproductive outcomes. Chronic inflammation, oxidative stress, and immune-mediated tissue remodeling impair endometrial receptivity, decidualization, and implantation, while dysregulated cytokine signaling may alter ovarian follicular development and oocyte competence [25,29]. These immune alterations provide a mechanistic link between endometriosis, infertility, and adverse obstetric outcomes.

Understanding the immune landscape of endometriosis has opened new avenues for therapeutic intervention. Immunomodulatory strategies targeting macrophage polarization, cytokine signaling, angiogenesis, and immune checkpoints are increasingly explored as adjuncts or alternatives to hormonal therapies [26,29]. Such approaches may be particularly relevant for patients with progesterone resistance or refractory disease.

### 1.4. Signaling Pathways

**Figure 2:**
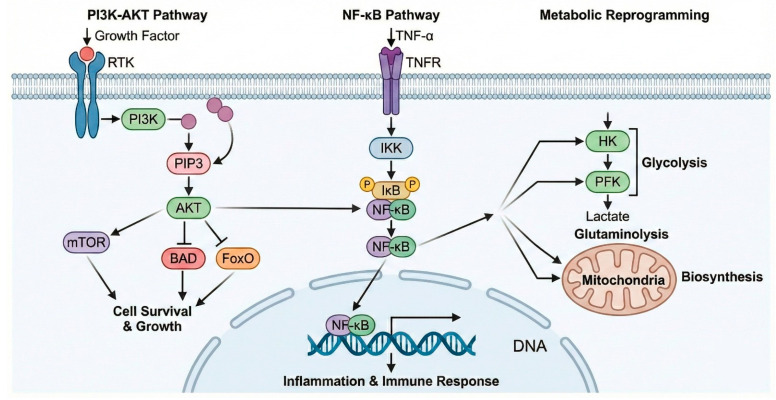
**Crosstalk between PI3K–AKT and NF-κB signaling pathways and metabolic reprogramming**. Left: Growth factor binding to receptor tyrosine kinases activates the PI3K–AKT signaling cascade, leading to mTOR activation and inhibition of pro-apoptotic factors (BAD and FoxO), thereby promoting cell survival and growth. Center: TNF-α signaling through TNF receptors (TNFRs) activates the IKK complex, resulting in IκB phosphorylation and degradation and subsequent nuclear translocation of NF-κB to induce inflammatory gene expression. Right: Crosstalk between PI3K–AKT and NF-κB signaling pathways promotes metabolic reprogramming, including upregulation of glycolytic enzymes (HK, PFK) and enhanced glutaminolysis, supporting mitochondrial biosynthetic activity, cellular proliferation, and inflammation. Arrows indicate activation or signaling flow, whereas T-bars indicate inhibition. Adapted from Refs. [8,22,23].

The major signaling pathways implicated in endometriosis, including PI3K–AKT and NF-κB signaling and their role in metabolic reprogramming, are schematically illustrated in Figure 2.

Endometriosis arises from complex interactions among genetic susceptibility, epigenetic remodeling, and environmental exposures, leading to sustained alterations in hormonal signaling, inflammatory responses, and cellular metabolism [8,10]. Key signaling pathways and molecular mediators involved in the pathogenesis of endometriosis are summarized in Table 1. Aberrant steroid hormone signaling, together with dysregulation of PI3K–AKT, TGF-β, MAPK, NF-κB, and mitochondrial pathways, drives lesion establishment, fibrosis, immune evasion, and defective decidualization [30,31,32].

Understanding these shared molecular mechanisms provides a strong rationale for targeted therapies and personalized reproductive management. Integration of multi-omic, metabolic, and immunopathological data enables the identification of biomarkers and therapeutic targets aimed at improving implantation success and reducing obstetric complications in women with endometriosis [33,34].

Endometriosis is an estrogen-dependent disorder characterized by progesterone resistance and aberrant steroid signaling, which underlies lesion growth, inflammation, and reproductive dysfunction [8,13]. Steroid hormones act through ERα, ERβ and PGR to regulate endometrial proliferation, differentiation, and receptivity [15]. Endometriotic stromal cells exhibit autonomous estradiol production via the steroidogenic pathway, aberrantly expressing enzymes such as StAR, CYP11A1, CYP17A1, and CYP19A1, in contrast to normal endometrial stromal cells [10].

Estrogen promotes endometrial proliferation and activates inflammatory mediators, including prostaglandin E_2_ (PGE_2_), thereby establishing a positive feedback loop that sustains lesion growth and chronic inflammation [8]. ERα and ERβ display distinct tissue distributions and transcriptional activities. In endometriotic stromal cells, ERβ expression is markedly increased, while ERα expression is reduced, contributing to estrogen dominance and progesterone resistance [8,17]. Enhanced ERβ signaling amplifies proliferative and inflammatory pathways, whereas reduced ERα impairs progesterone-mediated transcriptional responses, including retinoic acid (RA) synthesis and activation of HSD17B2, which is essential for estradiol inactivation [8].

Endometriosis depends on hormones, with estrogens and their receptors playing a central role in disease development [35]. Multiple estrogen receptors exist, notably estrogen receptor alpha (ERα) and estrogen receptor beta (ERβ), which act as nuclear transcription factors; ERα primarily controls genes involved in cell growth, while ERβ is more involved in regulating cell cycle progression and apoptosis [36].

The principal hormone driving the formation and maintenance of endometriotic lesions, and the associated pain and inflammation, is 17β estradiol (E2). Although E2 is largely synthesized locally within endometriotic tissue, it can also be delivered via the bloodstream; accumulation of E2 is believed to promote lesion progression through activation of estrogen receptors [37].

In normal endometrium, ERα levels exceed those of Erβ [37]. By contrast, women with endometriosis often show elevated ERβ expression in their eutopic endometrium, a pattern linked to greater inflammation, increased cellular proliferation, reduced apoptosis, and enhanced pain signaling [38]. Moreover, higher ERα expression in tissue has been associated with more severe symptoms, predicting deeper dyspareunia and moderate to severe dysmenorrhea, indicating that local receptor expression, in addition to circulating estrogen levels, relates to symptom severity [39].

Progesterone resistance in endometriotic stromal cells is further associated with defective PGR expression and disrupted paracrine signaling. Under physiological conditions, PGR activation induces RA production, which upregulates HSD17B2 in adjacent epithelial cells, promoting conversion of estradiol to the less active estrone. In progesterone-resistant endometriotic lesions, this pathway is compromised, resulting in persistent local estradiol accumulation and sustained inflammatory and proliferative signaling [40].

Beyond steroidogenic dysregulation, multiple intracellular signaling pathways contribute to endometriosis pathogenesis, reflecting the interplay between hormonal, inflammatory, and cell survival mechanisms. Key pathways include NF-κB, MAPK/ERK, PI3K/Akt/mTOR, Wnt/β-catenin, Rho/ROCK, TGF-β, and VEGF signaling, as well as pathways associated with oxidative stress and immune modulation [41]. These signaling networks collectively promote cellular proliferation, invasion, angiogenesis, fibrosis, and immune evasion, thereby supporting lesion persistence and progression.

Emerging evidence highlights a critical crosstalk between microRNAs (miRNAs) and the Wnt/β-catenin pathway in endometriosis. The Wnt/β-catenin pathway regulates proliferation, migration, epithelial–mesenchymal and endothelial–mesenchymal transition (EMT/EndMT), extracellular matrix remodeling, and stem-like properties, all hallmarks of endometriotic lesions [41]. Zang et al. (2024) demonstrated that dysregulated miRNAs directly or indirectly target core components of the Wnt/β-catenin pathway, including WNT ligands, Frizzled receptors, β-catenin, GSK3β, and downstream transcriptional effectors, thereby fine-tuning pathway activity [42]. miRNA-mediated modulation of Wnt/β-catenin signaling intersects with epigenetic and inflammatory pathways, reinforcing fibrotic remodeling and immune evasion within the lesion microenvironment. Several miRNAs implicated in endometriosis are responsive to estrogen signaling and inflammatory stimuli, suggesting that hormonal and inflammatory cues converge on miRNA networks to sustain Wnt-driven pathogenic programs [41,42].

Collectively, these interconnected mechanisms shape an estrogen-dominant, progesterone-resistant, inflammatory microenvironment that promotes fibrosis, altered decidualization, and infertility in women with endometriosis [10,23].

**Table 1:** **Key signaling pathways and molecular mediators involved in the pathogenesis of endometriosis**.

Pathway	Key Molecules/Genes	Functional Role in Endometriosis	Potential Therapeutic Targets/Interventions	References
Estrogen-mediated signaling	ERα, ERβ, CYP19A1	Promotes endometrial proliferation, survival of ectopic cells, estrogen dominance	Aromatase inhibitors, GnRH analogues, ERβ antagonists	[10,13]
Progesterone signaling	PGR, HSD17B2, RA	Regulates decidualization; progesterone resistance leads to impaired estradiol inactivation	Progestins (e.g., Dienogest), Retinoic acid modulators	[8]
NF-κB	RELA, IκBα	Mediates inflammation, oxidative stress, immune dysregulation	Antioxidants (e.g., Resveratrol, Curcumin), NF-κB inhibitors	[41]
MAPK/ERK	ERK1/2, MEK1/2	Cell proliferation, migration, invasion	Kinase inhibitors (MEK inhibitors)	[41]
PI3K/Akt/mTOR	PI3K, AKT, mTOR	Cell survival, anti-apoptosis, metabolic adaptation	mTOR inhibitors (e.g., Everolimus/Rapamycin - experimental), Metformin	[30,41]
Wnt/β-catenin	CTNNB1, FZD, LRP5/6	ECM remodeling, EndMT, cell migration	Wnt inhibitors, XAV939	[41]
TGF-β/Smad	TGF-β1, SMAD2/3/4	Fibrosis, myofibroblast activation, ECM deposition	Anti-fibrotic agents, TGF-β receptor kinase inhibitors	[41]
Rho/ROCK	RHOA, ROCK1/2	Cytoskeleton organization, motility, contractility	ROCK inhibitors	[40]
VEGF/Angiogenesis	VEGFA, VEGFR2	Promotes neovascularization, supports lesion survival	Anti-angiogenic agents	[41]
Oxidative stress & immune mediators	IL-6, TNF-α, NO, iron	Chronic inflammation, DNA damage, microenvironment remodeling	Iron chelators, Antioxidants (NAC, Vitamin C/E), Immunomodulators	[8]

This table summarizes the principal intracellular signaling pathways implicated in endometriosis, highlighting their key molecular components and functional roles in lesion establishment, persistence, and progression. Dysregulated estrogen and progesterone signaling, together with activation of inflammatory (NF-κB), proliferative (MAPK/ERK, PI3K/Akt/mTOR), fibrotic (TGF-β/Smad), migratory (Rho/ROCK), angiogenic (VEGF), and Wnt/β-catenin pathways, collectively contribute to estrogen dominance, progesterone resistance, chronic inflammation, fibrosis, immune evasion, and impaired decidualization. These interconnected networks underpin the complex molecular landscape of endometriosis and represent potential targets for biomarker discovery and therapeutic intervention.

## 2. Pathophysiological Context Relevant to Oocyte Longevity

### 2.1. Inflammation and Oxidative Stress in Endometriosis

#### 2.1.1. Inflammatory Cytokines and Granulosa Cell Dysfunction

Endometriosis creates a persistent, localized inflammatory environment in the peritoneal cavity and follicular fluid that undermines granulosa cell (GC) function and oocyte competence. Elevated pro-inflammatory cytokines, especially TNF-α, IL-1β, and IL-6, within the follicular microenvironment exert deleterious effects on ovarian physiology. TNF-α triggers apoptosis and suppresses steroidogenesis in GCs across species, acting as a negative regulator of GC function. Genetic ablation of TNF-α in mice increased GC proliferation and reduced oocyte apoptosis, extending fertility and supporting the idea that excessive TNF-α promotes follicular atresia and impairs follicular development. TNF-α can also provoke oxidative stress in oocytes, causing DNA and mitochondrial injury and reducing oocyte quality [43,44,45,46,47,48,49,50].

IL-1β downregulates FSH and LH receptor expression in GCs and reduces estradiol synthesis in rodent and human GCs, indicating an inhibitory influence on follicular maturation. Context-dependent effects have been reported: IL-1β stimulated bovine GC proliferation in one study and reduced apoptosis in rat follicular cells in another, but overall evidence supports a net negative impact of IL-1β on follicular development and oocyte maturation. IL-1 gene deficiency in mice was associated with increased fertility, reinforcing this interpretation [51,52,53,54,55].

IL-6 interferes with gonadotropin-driven differentiation by inhibiting FSH-induced LHR expression and suppressing FSH-stimulated steroidogenesis in several species. Although isolated reports describe enhancing effects, elevated IL-6 is commonly associated with aging and is generally detrimental to follicular development [56,57,58,59,60,61,62,63].

Chronic, low-grade inflammation fosters sustained oxidative stress within the ovary. Elevated ROS and reduced antioxidant capacity in follicular fluid correlate with poorer assisted reproductive technology (ART) outcomes. Maternal conditions such as PCOS, endometriosis, and advancing age further compromise the ovarian microenvironment and exacerbate oxidative and inflammatory insults [64,65,66,67].

#### 2.1.2. Immune Dysregulation and Macrophage Polarization

Endometriosis is consistently linked to chronic inflammation driven by recurrent bleeding from ectopic implants, which activates inflammatory and oxidative pathways and promotes adhesion formation, tissue remodeling, and fibrosis, processes that contribute to pain and infertility. Host susceptibility shapes the inflammatory phenotype: some women mount persistent responses that favor lesion survival. Microbial dysbiosis of the gut and reproductive tract has been implicated; altered microbiomes and estrobolome changes may promote lesion development, and fecal microbiota transfer or antibiotics can modulate lesion growth in models [8,48,68,69,70,71,72,73,74,75,76,77].

Immune surveillance is disrupted in endometriosis. Normally, macrophages clear refluxed menstrual debris; in endometriosis, repeated menstruation and inadequate clearance alter immune cell populations and cytokine profiles in peritoneal fluid. Macrophages polarize between pro-inflammatory M1 and reparative M2 phenotypes: early lesions show M1 predominance with enhanced NF-κB activation and production of VEGF, TNF-α, and IL-8, promoting estrogen-driven inflammation and implant proliferation. As lesions progress, M2 macrophages contribute to tissue repair and fibrogenesis via IL-10 and TGF-β. A Th2 skew underlies impaired immunosurveillance that allows ectopic tissue persistence; macrophages thus treat lesions as wounds, perpetuating inflammation, angiogenesis, and fibrosis [78,79,80,81,82,83,84,85,86].

#### 2.1.3. Iron Overload, Fenton Chemistry, and Ferroptosis

Recurrent intralesional hemorrhage uniquely intensifies oxidative stress in endometriosis: iron released from erythrocytes accumulates in the peritoneal cavity and cysts, driving Fenton chemistry and generating highly reactive species such as hydroxyl radicals and superoxide. Endometriotic cysts contain markedly elevated iron concentrations, and iron-driven autoxidation produces ROS that damage DNA and mitochondria, promote lipid peroxidation, and can trigger ferroptosis. Macrophage–lesion interactions may sometimes protect endometriotic cells from oxidative damage, enabling lesion persistence [87,88,89,90,91,92,93,94,95,96,97,98].

Free iron in endometrioma fluid is particularly harmful: concentrations are roughly ten times higher than serum and are elevated in adjacent follicular fluid. Ferrous iron catalyzes conversion of hydrogen peroxide to hydroxyl radicals via the Fenton reaction, producing one of the most damaging ROS. Excess iron and resultant ROS provoke inflammation, oxidative damage, lipid peroxidation, and ferroptotic cell death. Iron overload in follicular fluid has been shown to induce granulosa cell ferroptosis and oocyte dysmaturation in endometriosis models [93,94,95,96,97,98,99,100,101,102,103,104,105,106,107,108,109,110,111,112,113,114,115,116,117,118,119,120,121,122,123].

#### 2.1.4. Hypoxia, Metabolic Reprogramming, and Mitochondrial Adaptation

Hypoxia is central to lesion survival. Refluxed endometrial fragments face oxygen deprivation and reprogram metabolism away from oxidative phosphorylation toward glycolysis. Upregulation of glycolytic regulators such as PFKFB3, GLUT1, HK2, PDK1, and LDHA, together with HIF-1α and TGF-β, favors pyruvate and lactate production, supports proliferation, and reduces mitochondrial ROS generation. Activation of HK2 may divert glucose into the pentose phosphate pathway, generating NADPH and ribose for antioxidant defenses and DNA repair, an adaptation that supports lesion survival [100,101,102,103,104,105,106].

Mitochondrial dynamics, regulated fission and fusion mediated by MFN1/2 and DRP1, enable metabolic plasticity. Fusion enhances respiration and ATP production, supporting genomic stability and survival; fission and quality control permit removal of damaged organelles. Through modulation of mitochondrial morphology and turnover, endometriotic cells maintain redox homeostasis and adapt to hypoxia. ROS, hypoxia, and inflammation can also induce epigenetic changes that further enable survival strategies [107,108].

Endometriomas exert local toxic effects on the ovary. Follicular growth is suppressed in ovarian cortex adjacent to endometriomas, and women with endometriomas often yield fewer oocytes in ART, suggesting reduced ovarian responsiveness. Unlike other benign cysts, endometriomas lack a true capsule and are surrounded by stroma and a single epithelial layer, allowing toxic cyst contents, cytokines, ROS, and free iron to diffuse into adjacent ovarian cortex and follicles. Altered TNF-α, interleukin, and ROS levels in follicular fluid adjacent to endometriomas can induce oxidative stress, stromal fibrosis, and impaired folliculogenesis and oocyte maturation [3,104,109,110,111,112,113,114,115,116].

Ovarian fibrosis is a histologic hallmark associated with endometriomas: fibrosis within and around cysts correlates with reduced follicular density in adjacent cortex, likely reflecting impaired angiogenesis, increased atresia, and fibrotic remodeling. Disrupted iron homeostasis is known to induce fibrosis in other organs, and endometrioma-derived iron, cytokines, and ROS may synergize with TGF-β1 signaling to drive stromal fibrosis [117,118,119,120,121,122,123,124,125,126,127,128].

Whether endometriomas consistently reduce intrinsic oocyte quality remains debated. Some studies report poorer ART outcomes and oocyte abnormalities, zona pellucida hardening, spindle defects, and decreased mitochondrial number, while other reports find similar clinical pregnancy and live birth rates when multiple oocytes are retrieved and fertilized. Thus, endometriomas appear to reduce oocyte yield and follicular density, but the effect on the proportion of high-quality oocytes or embryos is inconclusive [103,110,111,112,113,114,115,116,117,118,119,120,121,122,123,124,125,126,127,128,129,130,131,132].

#### 2.1.5. Oxidative Stress Biomarkers and Clinical Heterogeneity

Studies document elevated oxidative stress markers, ROS, malondialdehyde (MDA), nitric oxide (NO), and 8-OH-2′-deoxyguanosine (8-OHdG), in peritoneal fluid, follicular fluid, serum, and granulosa cells of women with advanced endometriosis, though findings vary across cohorts and methods. Heterogeneity in patient selection, disease classification, prior treatments, and analytic methods likely contributes to inconsistent results [133,134,135,136,137,138,139].

Sirtuins, NAD+-dependent deacetylases, act as metabolic sensors responding to inflammatory and oxidative cues and are implicated in reproductive aging and stress responses. SIRT1, SIRT3, and SIRT5 are expressed in granulosa and cumulus cells and play roles in oogenesis. SIRT3, predominantly mitochondrial, supports mitochondrial homeostasis and antioxidant defenses via SOD2 activation. Clinical data indicate elevated SIRT3 in PBMCs from patients with deep endometriosis compared with ovarian endometrioma, suggesting an association between SIRT3 expression and disease severity or systemic impact; elevated SIRT3 may reflect a compensatory antioxidant response [140,141,142,143,144,145].

The epithelial–mesenchymal transition (EMT) contributes to endometriosis pathobiology. Patients display elevated serum IL-6, IL-8, and IL-12 and reduced IL-10, alongside increased EMT markers (cadherins, TGF-β and HIF-1α) in serum and follicular fluid; these signatures correlate with fewer good-quality embryos and can discriminate affected patients. Serum IL-8 and E-cadherin, together with follicular fluid IL-10, have been reported to predict positive IVF outcomes in one study [146].

#### 2.1.6. Therapeutic Implications and Future Perspectives

Preclinical interventions targeting redox and inflammatory pathways validate the pathogenic importance of the inflammation–oxidative axis. Nanocomposite approaches combining polydopamine (for ROS scavenging and macrophage repolarization) with dydrogesterone have shown lesion targeting, ROS reduction, decreased inflammatory cytokines, macrophage repolarization away from pro-inflammatory phenotypes, and lesion atrophy without compromising ovarian reserve in animal models. Strategies that selectively increase oxidative stress within lesions to induce ROS-mediated apoptosis or ferroptosis, or that block HMGB1/TLR4–NF-κB signaling in granulosa and stromal compartments, can reduce lesion viability and ameliorate inflammation-driven infertility in experimental systems. Pharmacologic modulation of iron handling and ferroptosis pathways is promising given the centrality of iron-driven ROS in lesion biology [147,148].

Agents that restore antioxidant enzyme activity show efficacy in preclinical models: for example, ramipril restored catalase activity, reduced serum NO and tissue MDA, decreased VEGF expression, and induced apoptotic signaling (cleaved PARP1, increased Bax:Bcl-2 ratio, cleaved caspase-3) in an endometriotic rat model. Subsequent administration of nitric oxide donors reversed these effects, underscoring the mechanistic link between redox modulation and lesion survival; detailed mechanisms warrant further study [149].

Clinical evidence linking oxidative stress markers to disease phenotype is mixed. Some studies report higher systemic oxidative stress in deep endometriosis compared with ovarian endometrioma and suggest prior hormonal treatment may modulate oxidative markers favorably. Metabolomic analyses have identified distinct follicular fluid profiles in more severe disease, though redox markers do not always differ significantly across phenotypes, likely reflecting heterogeneity in prior treatments, surgical histories, and study designs [150].

Collectively, the literature supports a feed-forward model in which inflammation and oxidative stress reciprocally reinforce one another: oxidative injury promotes DAMP release and cytokine production, which sustain ROS generation and immune activation, enabling ectopic endometrial cells to survive, proliferate, and impair reproductive function. This integrated perspective explains lesion heterogeneity, chronic pelvic pain, reduced ovarian reserve and follicular density adjacent to endometriomas, and variable ART outcomes. It also highlights actionable therapeutic targets: iron chelation and ferroptosis modulation, antioxidant and redox-modulating therapies, NF-κB/COX-2 blockade, inhibition of neurotrophic and angiogenic factors, and antifibrotic strategies, each meriting further clinical evaluation as adjuncts to surgical and hormonal approaches aimed at preserving fertility and alleviating symptoms [139].

### 2.2. Cellular Aging, Redox Balance, and the Autophagy System

#### 2.2.1. Sirtuins and Mitochondrial Integrity

Aging-related cellular changes critically shape the redox balance and reproductive competence in endometriosis. Sirtuins, NAD+-dependent deacetylases, act as metabolic sensors that respond to oxidative and inflammatory cues and are tightly linked to aging and reproductive decline [151]. Among them, mitochondrial SIRT3 is especially relevant because of its role in preserving mitochondrial integrity and bolstering antioxidant defenses through the activation of SOD2, thereby enhancing the removal of ROS and supporting mitochondrial homeostasis [152,153]. Because fertility is highly sensitive to cumulative oxidative stress and metabolic dysregulation, perturbations in sirtuin signaling can compromise oocyte quality and developmental potential.

Clinical data indicate that SIRT3 expression is elevated in PBMCs from patients with more severe, deep endometriosis compared with ovarian-limited disease. This increase, particularly evident in individuals with prior surgical treatment, correlates with higher SOD2 levels, suggesting a systemic, possibly compensatory antioxidant program linked to disease burden and surgical history [139]. Experimental models further support a protective role for SIRT3 in gametes: SIRT3 activation reduces oocyte ROS, preserves spindle integrity, and improves embryo development in mouse and human systems, and SIRT3 upregulation in metabolic stress models restores oocyte competence [154]. Collectively, these observations imply that age-associated declines in mitochondrial resilience and sirtuin-mediated defenses contribute to the vulnerability of reproductive cells in endometriosis, while modulation of the SIRT3→FOXO3a→SOD2 axis represents a promising avenue to counteract oxidative aging in the ovarian microenvironment.

#### 2.2.2. Ovarian Aging and Autophagic Dysfunction

In mammals, including humans, ovarian aging results in both a reduction in oocyte number and a deterioration of oocyte competence, contributing to increased infertility and embryonic abnormalities. Quercetin promotes in vitro maturation of oocytes from humans and aged mice [154]. With advancing age, oocytes progressively accumulate oxidative damage, including protein and lipid peroxidation, and mitochondrial dysfunction becomes evident as early as the primordial follicle. Although mitochondria are the primary energy source for oocytes, they are also major generators of ROS. In aged ovaries, diminished antioxidant defenses exacerbate this dysfunction, forcing oocytes to rely increasingly on glycolysis due to the depletion of essential metabolites such as NAD+ [155].

This age-related oxidative stress is compounded by the accumulation of Rubicon, an endogenous autophagy inhibitor. Beyond suppressing autophagy, Rubicon interacts with the p22phox subunit of the NADPH oxidase complex, promoting ROS generation and the release of pro-inflammatory mediators like TNF-α and IL-6 [156]. Furthermore, oxidative stress downregulates SIRT1, a critical regulator of cellular stress responses [155]. The loss of SIRT1 facilitates Rubicon’s association with the PI3K complex, impairing autophagosome maturation [157]. Experimental interventions, such as treatment with trehalose, have been shown to reduce DNA damage by lowering Rubicon levels [158], while quercetin enhances resistance to oxidative injury by activating the Nrf2 pathway [159]. These findings highlight the Beclin1–Rubicon complex as a central mechanism in oxidative stress-induced cytotoxicity.

Autophagy plays a pivotal role in infertility disorders, although its precise contribution remains complex due to assessment challenges [160]. In endometriosis, chronic pelvic inflammation disrupts the ovarian microenvironment and reduces reserve, a condition often worsened by surgical excision [161]. Granulosa cells from these patients exhibit elevated markers of DNA damage (e.g., 8-OHdG), inflammatory cytokines (IL-1β, IL-6), ER stress, and apoptosis [162]. Additionally, oxidative stress-mediated downregulation of EZH2 upregulates IL1R2, suppressing ovulatory signaling [163].

Paradoxically, autophagy appears upregulated in endometriosis granulosa cells (increased Beclin1, elevated LC3-II/LC3-I), which correlates with increased expression of steroidogenic enzymes [164]. This activity is crucial for progesterone synthesis [165]. However, high progesterone levels in endometriotic cysts may suppress autophagy in the uterine endometrium [166]. The scaffold protein FIP200 is also essential for progesterone receptor signaling; its loss leads to decidualization failure [167]. Future diagnostic strategies may focus on proteomic profiling to distinguish between disorders of suppressed autophagy, like POI, marked by Rubicon, and excessive activation, like endometriosis, marked by Beclin1.

#### 2.2.3. Senescence and Decidualization Failure

Key of cellular processes, autophagy, decidualization, and senescence, are disrupted in endometriosis, largely through alterations in the p53–AMPK–mTOR signaling axis [168,169,170,171], impairing endometrial receptivity and contributing to infertility [172,173]. Normal decidualization requires coordinated estrogen and progesterone signaling [174,175,176,177] and transcription factors like FOXO1, which induce p21/p27 for essential cell cycle arrest and physiological senescence [178,179]. However, endometriosis is characterized by progesterone resistance, reduced PGR, impaired non-genomic signaling, and estrogen excess, which suppress FOXO1 and HOXA10 [180]. This failure is exacerbated by inflammatory cytokines (TNF-α, IL-1), WNT/β-catenin abnormalities, NEK2-mediated FOXO1 destabilization [35], and aberrant NF-κB and PI3K/AKT signaling [179,181,182].

While senescence is normally transient during decidualization and cleared by apoptosis, endometriosis leads to the accumulation of aberrant, pathological senescent cells. This accumulation is driven by p53 dysfunction (MDM2 overexpression) [183,184,185] and chronic ROS/iron metabolism abnormalities that activate p53–p21 [186,187], promoting markers like p16 and SA-β-gal [188]. These cells secrete a Senescence-Associated Secretory Phenotype (SASP) containing ILs, chemokines, and MMPs [185,189,190,191]. Under physiological conditions, SASP recruits uterine NK cells for clearance, but in endometriosis, NK cell number and function are reduced due to aberrant IL-15 and CXCL12 expression [192]. Consequently, the persistent SASP amplifies inflammation, fibrosis, pain, and infertility [185,189].

### 2.3. Follicular Microenvironment Disruption

Ovarian endometriomas disrupt the follicular microenvironment through iron-mediated oxidative stress, granulosa cell dysfunction, including altered aromatase and miRNA-mediated IGF1R suppression, cortical fibrosis with reduced follicle density, and measurable declines in mature oocyte yield during IVF, all supported by recent histological, biochemical, molecular and clinical studies [193].

miRNA-mediated granulosa cell dysfunction. Recent molecular profiling of follicular fluid demonstrates upregulation of let 7 family microRNAs in ovaries affected by endometriomas, with functional evidence that let 7 targets IGF1R, reducing granulosa cell proliferation, steroidogenic capacity, ATP production and increasing oxidative stress, mechanisms that directly impair oocyte support and maturation [194].

Intrafollicular iron excess correlates with an increased reactive oxygen species, depleted antioxidant defenses (notably glutathione pathways), and altered mitochondrial respiration in follicular cells; experimental and translational work suggests these changes reduce oocyte competence and may contribute to poorer embryo development in some cohorts [195].

Large retrospective and cohort studies show that women with ovarian endometriomas often produce fewer mature (metaphase II) oocytes during controlled ovarian stimulation, even when gross oocyte morphology scores are similar to controls; Robin et al. (2021) reported a statistically significant reduction in MII yield in endometriosis patients undergoing IVF ICSI [6].

Endometrioma-associated inflammation alters local cytokine and growth factor signaling; granulosa cells from affected follicles show reduced aromatase expression and estradiol synthesis, increased apoptotic markers, and dysregulated paracrine signaling that together compromise oocyte maturation and follicular competence [196].

Taken together, iron-mediated oxidative stress, miRNA-driven suppression of IGF signaling, structural cortical damage, and granulosa cell steroidogenic failure form a coherent pathophysiological cascade linking endometriomas to reduced ovarian reserve and lower mature oocyte yield; these mechanisms support targeted strategies (antioxidant modulation, iron chelation research, miRNA pathway modulation, and surgical conservative decision-making) to preserve fertility and improve ART outcomes [197].

### 2.4. Metabolic and Nutrient-Sensing Pathways

#### 2.4.1. Systemic Metabolic Signatures

Endometriosis is characterized by a reproducible systemic metabolic disturbance, marked by elevated ketone bodies, amino acid imbalances, disrupted one-carbon metabolism, and cell type-specific metabolic reprogramming that links nutrient sensing to inflammation, oxidative stress, and proliferative lesion behavior [198].

Endometriosis patients show altered circulating metabolites consistent with a shift toward alternative energy substrates and nitrogen redistribution: a targeted serum metabolomics study using ^1^H NMR reported increased β-hydroxybutyrate and glutamine and decreased tryptophan, together with perturbations in nitrogen metabolism, pyrimidine metabolism, glutamine–glutamate metabolism, and aminoacyl tRNA biosynthesis, supporting the idea that systemic metabolite changes reflect lesion biology and may serve as non-invasive biomarker candidates. The authors emphasized that this metabolomic strategy can discriminate patients with painful endometriosis from controls, identified specific metabolites driving group separation, and proposed follow-up work to test effects of hormonal therapy and NSAIDs, as well as to compare symptomatic versus asymptomatic cases, while noting that small sample sizes require cautious interpretation [199].

Complementing serum metabolomics, clinical biochemical profiling reveals disturbances in one-carbon and methylation-related nutrients: a recent Molecular Biology Reports study found elevated homocysteine and significantly lower folic acid and vitamin B12 in endometriosis cases versus controls, a pattern that can exacerbate oxidative stress, impair DNA/histone methylation, and amplify inflammatory signaling, thereby linking metabolic imbalance to immune dysfunction and systemic disease manifestations. These metabolic alterations occur alongside dysregulated cytokine networks, and the authors highlight the involvement of canonical inflammatory signaling axes, NF-κB, JAK STAT, and TGF β/SMAD, as mediators that connect metabolic state to cytokine expression and that may represent actionable anti-inflammatory therapeutic targets [200].

At the tissue and cellular level, single-cell transcriptomics demonstrates cell type-specific metabolic reprogramming within endometriotic lesions: paired analyses of eutopic and ectopic endometrium revealed that perivascular, stromal, and endothelial cells in ectopic lesions exhibit the largest shifts in metabolic pathway expression, with pronounced changes in AMPK signaling, HIF-1 signaling, glutathione metabolism, oxidative phosphorylation, and glycolysis. Notably, the study documents transcriptomic co activation of glycolytic and oxidative programs in perivascular and stromal cells, a hybrid metabolic phenotype that supports lesion survival, angiogenesis, and repair and that nominates perivascular compartments as promising non-hormonal therapeutic targets [201].

Together, these complementary lines of evidence, serum metabolomics, systemic nutrient/methylation profiling, and single-cell metabolic mapping, converge on a model in which metabolic dysregulation both reflects and drives inflammation, oxidative stress, and proliferative lesion behavior, offering a coherent framework for biomarker development and for testing metabolic or anti-inflammatory interventions in endometriosis [20].

#### 2.4.2. Nutrient Sensing and Longevity Pathways

Reproductive longevity is tightly linked to systemic metabolic status. Nutrient-sensing pathways such as insulin/IGF-1, mTOR, AMPK, and sirtuins integrate energy balance with ovarian function. In endometriosis, metabolic dysregulation intersects with inflammatory signaling, accelerating ovarian aging. Excess caloric intake and insulin resistance activate mTOR, promoting proliferation and inflammatory cytokine production. Conversely, AMPK activation supports mitochondrial biogenesis and reduces inflammation, with experimental models showing restoration of ovulatory function. Sirtuins regulate mitochondrial function and chromatin remodeling, and their activation has been linked to extended ovarian lifespan. Lifestyle interventions, anti-inflammatory diets rich in omega 3 fatty acids, fiber, and polyphenols, modulate these pathways, reducing mTOR activity and supporting AMPK signaling. These findings suggest that metabolic regulation is a modifiable axis of reproductive longevity in endometriosis [202,203].

#### 2.4.3. Glycolytic Reprogramming (Warburg Effect)

Endometriotic cells adapt to hypoxia and inflammation by shifting metabolism toward glycolysis. HIF-1α stabilizes and induces GLUT1, HK2, LDHA [204,205]. Mitochondrial oxidative phosphorylation is reduced, while glycolytic enzymes (HK2, PKM2 and PFKFB4) are upregulated [206,207]. Non-human primate models confirm impaired oxidative phosphorylation. This metabolic reprogramming resembles the Warburg effect. Estrogen and PI3K/AKT/mTOR signaling further enhance glycolysis [206,208]. Cytokines (IL-6, TNF-α) activate NF-κB and STAT3, promoting fibrosis and glycolysis [209,210,211]. NF-κB directly regulates glycolytic genes. Lactate accumulation induces histone lactylation, linking metabolism to epigenetic regulation [212].

### 2.5. Iatrogenic Factors

Clinical management of endometriosis can unintentionally shorten reproductive lifespan: surgical excision of endometriomas and some hormonal regimens reduce ovarian reserve, while tailored conservative surgery, perioperative hormonal strategies, and early fertility-preservation counseling (oocyte cryopreservation) can help preserve fertility longevity [213].

Ovarian endometrioma surgery commonly removes adjacent healthy ovarian cortex, producing reduced follicular density and an accelerated decline in ovarian reserve; contemporary analyses identify factors that predict reserve loss after laparoscopic cystectomy, including cyst size, bilaterality, and the surgical technique used [214].

Systematic appraisals show that the presence of an endometrioma itself is associated with lower baseline ovarian reserve and reproductive potential. Key determinants of post-operative reserve include whether the procedure is unilateral or bilateral and the extent of inadvertent cortical removal during stripping [214].

Randomized controlled data indicate that perioperative hormonal modulation influences ovarian reserve trajectories: a randomized trial comparing perioperative GnRH agonist versus dienogest found that pre- and post-operative hormonal suppression can modify AMH changes after cystectomy, suggesting that short, targeted hormonal regimens may mitigate immediate reserve loss, although long-term effects on ovarian longevity remain incompletely defined [215].

Meta-analyses comparing surgical approaches report that cystectomy (stripping) tends to reduce AMH more than ablative or combined excisional/ablative techniques, while ablation may preserve more ovarian tissue at the cost of higher recurrence; thus, choice of technique requires balancing recurrence risk against fertility longevity. A multicenter randomized trial comparing stripping with combined excisional/ablative approaches further supports that less aggressive tissue removal can reduce the impact on ovarian reserve without clearly improving outcomes [216].

Given these trade-offs, recent clinical guidance emphasizes fertility-preserving decision making: for young patients with severe disease or bilateral lesions, clinicians should discuss the risks of immediate surgery versus conservative management and offer fertility preservation options (oocyte or embryo cryopreservation) before cystectomy when future childbearing is a priority. Evidence syntheses and practice reviews conclude that oocyte cryopreservation is the most established fertility-preservation strategy in endometriosis, with accumulating data showing acceptable oocyte quality and satisfactory ART outcomes when sufficient oocytes are banked prior to surgery or disease progression [217].

Emerging recommendations advocate a proactive, individualized pathway to maximize fertility longevity: early AMH/AFC assessment, multidisciplinary counseling, consideration of conservative surgical techniques (combined excision/ablation or careful hemostasis to spare cortex), selective perioperative hormonal protocols, and timely referral for oocyte cryopreservation for patients at high risk of reserve loss. Implementing this model aims to preserve reproductive lifespan while still addressing pain and disease control, aligning therapeutic efficacy with long-term fertility goals.

**Figure 3:**
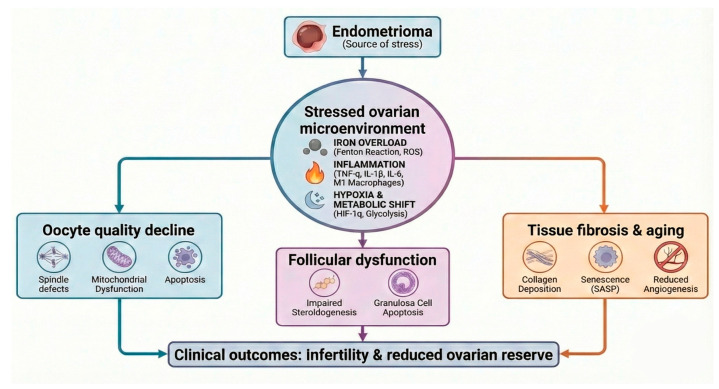
**Pathophysiological impact of endometrioma on the ovarian microenvironment and reproductive outcomes**. The presence of an endometrioma serves as a continuous source of stress, triggering a complex cascade within the ovarian niche. This “stressed microenvironment” is characterized by: (i) Iron overload, which promotes the Fenton reaction and the accumulation of ROS; (ii) Chronic inflammation, mediated by pro-inflammatory cytokines (TNF-α, IL-1β and IL-6) and M1 macrophage polarization; and (iii) Hypoxia, leading to an HIF-1α-driven metabolic shift toward glycolysis. These stressors converge into three deleterious pathways: Oocyte quality decline, Follicular and Tissue fibrosis and. The synergy of these mechanisms ultimately leads to the clinical hallmarks of endometriosis-associated subfertility: infertility and a significantly reduced ovarian reserve. Adapted from [4,8,10,15,17].

Together, inflammation, oxidative stress, metabolic reprogramming, immune dysregulation, and aging-related pathways converge to disrupt the follicular microenvironment, impair granulosa cell support, and compromise oocyte quality and longevity, as schematically illustrated in Figure 3.

## 3. Morphological and Ultrastructural Alterations in Oocytes

Large-cohort morphology scoring largely fails to capture the functional frailty of oocytes from women with endometriosis; focused studies show reproducible microstructural and bioenergetic defects (granularity, vacuoles, zona changes, spindle instability in some series, and mitochondrial loss/mtDNA depletion) that plausibly explain reduced MII yield, embryo numbers, and pregnancy rates [218].

### 3.1. Morphology: Cohort Neutrality Versus Targeted Vulnerability

Morphological abnormalities tend to be distributed across the cohort and should be interpreted as phenotypic manifestations of a common biological context rather than as markers of intrinsic incompetence. This interpretation is consistent with the limited reproducibility and weak predictive value of oocyte morphological grading, as well as with clinical evidence showing that morphologically suboptimal oocytes can still give rise to euploid embryos and successful pregnancies [219].

In contrast, the targeted vulnerability model assumes that certain oocytes are intrinsically more fragile and that morphological abnormalities reflect specific cellular damage, such as meiotic errors, mitochondrial dysfunction, or maturational asynchrony. Within this framework, morphology would serve as a proxy for biological competence and could theoretically support selective exclusion. However, evidence supporting this model remains inconsistent and appears to be restricted to rare and severe morphological alterations [220].

Overall, available data predominantly support the cohort neutrality paradigm, particularly for common cytoplasmic abnormalities such as granularity, vacuolization, and zona pellucida alterations, which show limited and inconsistent associations with oocyte developmental competence [220]. Only a limited subset of severe or rare morphological defects appears to reflect true targeted vulnerability and may be associated with impaired fertilization or developmental potential [221].

In summary, oocyte morphology appears to reflect the biological state of the cohort more than the fate of individual oocytes, with most observed abnormalities representing phenotypic variability within a shared environment rather than selective cellular vulnerability [222].

It has been hypothesized that morphological defects observed, including cytoplasmic granularity and/or the presence of vacuoles, could affect fertilization; however, the predictive value of these observations is limited due to the restrictions of non-invasive techniques such as simple transmitted light microscopy and the problem of subjectivity in the morphological evaluations. Moreover, morphology itself could be influenced by other factors, such as the ovarian stimulation or the hormonal milieu; therefore, its potential as a predictive factor of clinical outcome needs further investigation [222].

Some authors conducted functional studies comparing immature oocytes obtained from women with endometriosis to those from control patients without endometriosis, irrespective of infertility etiology. They reported that oocytes from women with endometriosis exhibited increased cortical granule loss and zona pellucida (ZP) hardening, alterations that may interfere with fertilization, ZP dissolution, and the embryo’s ability to undergo hatching and implantation [223].

Furthermore, the developmental competence of these immature oocytes was assessed through in vitro maturation (IVM). A significantly lower proportion of germinal vesicle (GV) and metaphase I (MI) oocytes from the endometriosis group progressed to the metaphase II (MII) stage compared with controls [224].

In line with these functional findings, a retrospective study evaluating oocyte morphology in endometriosis patients reported a higher incidence of dark central granulation of the ooplasm in endometriosis-positive cycles. More recently, Borges and colleagues observed a significant increase in extra-cytoplasmic, but not intra-cytoplasmic, oocyte abnormalities in patients with endometriosis. In this study, endometriosis was negatively correlated with embryo developmental potential, although blastocyst formation rates were not affected; however, no data on blastocyst quality were provided [225].

Large retrospective and registry-style analyses report no consistent shift in composite morphology scores (AOQI/MOMS) despite worse clinical outputs (fewer MII oocytes, fewer embryos, lower cumulative pregnancy/implantation), a pattern compatible with subtle rather than overt morphological damage [6]. By contrast, focused comparisons and meta-analyses identify higher rates of cytoplasmic granularity, intracytoplasmic vacuoles, altered perivitelline space, and zona pellucida hardening in endometriosis, especially when follicles are adjacent to endometriomas or when lab assessment is optimized to detect fine dysmorphisms [115].

### 3.2. Spindle and Chromosomal Resilience: Mixed but Mechanistically Plausible

Controlled clinical assessments using polarized light sometimes find no difference in spindle visibility between endometriosis and tubal-factor controls, yet experimental and IVM-based work repeatedly show increased spindle abnormalities and chromosomal misalignment under endometriosis-associated follicular fluid or oxidative conditions. This heterogeneity likely reflects disease stage, follicular microenvironment, and methodological sensitivity; spindle disruption is real in some contexts and may contribute to aneuploidy risk or failed fertilization in susceptible oocytes [226].

The meiotic spindle is essential for accurate chromosome alignment and segregation, and its disruption may result in impaired fertilization and aneuploidy. In ICSI cycles, oocytes with a visible and normally organized spindle show higher fertilization and embryo euploidy rates. Although polarized light microscopy allows non-invasive spindle visualization and has been proposed as a marker of oocyte quality, evidence supporting its clinical relevance in women with endometriosis remains inconsistent [115].

Most studies have evaluated spindle morphology in oocytes obtained through in vitro maturation (IVM) protocols. While some reports found no significant differences in spindle integrity or visibility between endometriosis patients and controls, others observed reduced fertilization rates in moderate to severe disease or a higher incidence of abnormal spindles in endometriosis. Notably, the only study assessing spindle morphology in mature oocytes did not detect significant alterations associated with endometriosis, highlighting the limitations of extrapolating findings from IVM-derived oocytes [115].

Overall, current data are inconclusive, and further studies focusing on mature oocytes are required to clarify the value of spindle assessment as a marker of oocyte quality in women with endometriosis.

### 3.3. Cytoplasmic Ultrastructure and Mitochondrial Compromise

Transmission electron microscopy and molecular assays converge on a bioenergetic deficit: oocytes from women with endometriosis show fewer mitochondria, higher rates of swollen/abnormal mitochondria, and reduced mtDNA copy number, without consistent changes in Golgi or cortical granule architecture in every study. These mitochondrial deficits map well to reduced maturation competence, impaired fertilization, and early developmental arrest because ATP supply and calcium handling are central to meiotic completion and embryo cleavage [227].

Cytoplasmic composition is a critical determinant of oocyte competence and early embryo development [228]. Mature human oocytes are characterized by an exceptionally high mitochondrial content, reaching up to 10^5^ mitochondria per cell. Mitochondrial abnormalities or dysfunction, well described in association with maternal aging, are known to adversely affect fertilization and embryonic development. Despite this, the impact of endometriosis on oocyte cytoplasmic ultrastructure has been poorly investigated [115].

To date, the only study addressing this aspect was conducted by Xu et al. in the context of ICSI cycles, using transmission electron microscopy to analyze 50 MII oocytes from women with laparoscopically diagnosed minimal/mild endometriosis and controls with tubal or male factor infertility [4]. Oocytes from women with endometriosis showed altered nuclear features, including decentralized chromatin and enlarged nucleoli, as well as a higher proportion of abnormal mitochondria and an overall reduced mitochondrial number. This finding was corroborated by a significantly lower mitochondrial DNA (mtDNA) copy number in oocytes from endometriosis patients compared with controls, as assessed by quantitative real-time PCR [131].

### 3.4. Mechanisms That Link Microdamage to Clinical Outcomes

The most consistent mechanistic threads are oxidative stress, inflammatory cytokines, and granulosa cell dysfunction in the follicular milieu. Oxidative markers in follicular fluid correlate with spindle and mitochondrial abnormalities; granulosa cell transcriptomic and secretory changes can reduce metabolic support to the oocyte, amplifying vulnerability during stimulation and fertilization [229].

## 4. Clinical Outcomes and Embryo Development in Endometriosis

Mechanisms associated with endometriosis—oxidative stress, iron overload, altered cytokine signaling, dysregulated miRNA networks, and epigenetic modifications—can each plausibly impair embryonic development at distinct stages and thereby influence clinical outcomes [14,15,16,17,18,19,20,41,42]. Oxidative injury in the follicular microenvironment (elevated ROS, lipid peroxidation, 8-OH-dG) can compromise oocyte cytoplasmic maturation, mitochondrial integrity, and spindle stability, producing embryos with reduced developmental competence or, in some settings, an increased risk of aneuploidy [50,133,134,135,136,137,138,139]. Iron-driven Fenton chemistry within endometriomas and the peritoneal cavity generates highly reactive hydroxyl radicals that damage DNA and membranes; when follicular fluid adjacent to endometriomas is iron-loaded, experimental data link granulosa-cell dysfunction and ferroptotic processes to oocyte dysmaturation and poorer embryo metrics [93,94,95,96,117,118,119,120,121,122,123].

Altered cytokine milieus—characterized by increased IL-6, TNF-α, and IL-8 and decreased IL-10—and chronic inflammation perturb cumulus–oocyte signaling and steroidogenic support. These disturbances can impair meiotic progression and early zygotic gene activation, reducing cleavage competence or blastocyst quality in some cohorts [43,44,45,46,56,57,58,59,60,61,62,63,146]. Concurrently, miRNA dysregulation and epigenetic remodeling in follicular cells and the endometrium may modify maternal transcripts and secreted factors that shape embryo metabolism and implantation potential. miRNA-mediated modulation of pathways such as Wnt/β-catenin, PI3K–AKT, and TGF-β can therefore translate molecular alterations into altered embryo phenotype or impaired endometrial receptivity [8,14,15,16,17,18,19,20,41,42].

Clinical correlations are heterogeneous because the magnitude, timing, and anatomical localization of these insults vary. Local ovarian toxicity (for example, iron/ROS exposure adjacent to an endometrioma) is more likely to reduce oocyte yield and to affect nearby follicular cohorts, whereas systemic or superficial disease may exert subtler effects via circulating cytokines or altered endometrial receptivity [109,110,111,112,113,114,115,116,117,118]. Accordingly, mechanistic markers map onto different clinical endpoints: iron/ROS signatures and granulosa-cell mitochondrial defects more directly predict oocyte yield, morphology, and early embryo development (fertilization, cleavage, blastulation), while miRNA/epigenetic and cytokine profiles may better predict implantation success and pregnancy maintenance (implantation, clinical pregnancy, live birth) [41,42,50,110,111,112,113,114,115,129,130,131,132,133,134,135,136,137,138,139].

To strengthen causal inference in future studies, we recommend integrating mechanistic biomarkers with clinical embryo endpoints: pair follicular fluid and granulosa cell assays (ROS, MDA, 8 OHdG, iron, SOD2/SIRT3 expression) with single oocyte assessments (spindle imaging, mitochondrial copy number, transcriptomics) and standardized embryo culture/readouts (morphokinetics, euploidy). Nested analyses that relate specific molecular perturbations to discrete outcomes (oocyte maturation, fertilization, blastocyst quality, implantation, live birth) will clarify which mechanisms are most clinically consequential and when interventions (antioxidants, iron chelation, metabolic modulators) are likely to improve reproductive endpoints [123,133,134,135,136,137,138,139,147,148,149].

### 4.1. Fertilization Rates

Fertilization outcomes vary by insemination method and disease context. Comparative analyses indicate that conventional IVF and ICSI may yield different fertilization efficiencies in patients with endometriosis, with ICSI often mitigating some fertilization deficits attributed to altered follicular or sperm–oocyte interactions; Robin et al. (2021) report lower overall fertilization metrics in endometriosis cohorts but show that ICSI can partially normalize fertilization rates compared with standard IVF [6]. Clinically, this supports selective use of ICSI when prior fertilization failure or severe endometriosis-related follicular dysfunction is suspected.

### 4.2. Cleavage-Stage Embryo Quality

Endometriosis is associated with reduced proportions of high-grade cleavage-stage embryos. Meta-analytic evidence synthesizing multiple IVF cohorts demonstrates a statistically significant reduction in the rate of high-grade cleavage embryos among women with endometriosis compared with controls, suggesting compromised early embryonic competence likely driven by altered oocyte quality and follicular microenvironment perturbations [115]. These findings align with mechanistic data implicating oxidative stress, iron overload, and granulosa-cell dysfunction as upstream contributors to poorer cleavage-stage morphology.

### 4.3. Blastocyst Formation and Euploidy

Blastulation rates may be modestly reduced, but aneuploidy rates appear largely unaffected by endometriosis. Recent reports indicate that while progression to blastocyst can be lower in some endometriosis cohorts, reflecting earlier compromises in oocyte competence and embryo developmental kinetics, chromosomal competence as measured by euploidy does not show a consistent increase in aneuploidy attributable to endometriosis per se. A 2025 Scientific Reports abstract indicates that aneuploidy rates were not significantly different between endometriosis and control groups, implying that reduced blastulation is more likely due to cytoplasmic or metabolic defects rather than gross chromosomal errors [230].

Miscarriage risk and RSA: Endometriosis is linked to an elevated risk of miscarriage in some studies, with immune and inflammatory dysregulation proposed as mediators. Recent analyses and registry data associate endometriosis with higher rates of early pregnancy loss and recurrent spontaneous abortion; mechanistic work implicates altered uterine immune tolerance, aberrant cytokine profiles, and impaired decidualization as contributors. A 2025 JARG report highlights the association between endometriosis and RSA and emphasizes immune dysfunction as a plausible causal pathway [9].

The pattern—reduced fertilization efficiency (partially remediable by ICSI), fewer high-grade cleavage embryos, lower blastulation in some cohorts, but no clear increase in embryo aneuploidy—points to oocyte cytoplasmic and metabolic impairment (oxidative stress, mitochondrial dysfunction, altered granulosa support) rather than primary chromosomal instability as the dominant driver of embryologic deficits.

### 4.4. Clinical Heterogeneity and Contradictory Evidence

Clinical data on whether endometriosis directly alters oocyte morphology and on its impact on ART outcomes are heterogeneous and sometimes apparently contradictory. Several studies report increased zona pellucida abnormalities, cytoplasmic granularity, spindle defects, and lower implantation rates for oocytes from affected women or for embryos derived from them [110,111,112,113,114,115,129,130,131]. By contrast, other well-controlled ART series—especially those that match or adjust for age, ovarian reserve, stimulation protocol, and oocyte yield—report comparable fertilization, blastulation, euploidy, clinical pregnancy, and live birth rates between women with and without endometriosis [111,132]. These divergent findings are not necessarily mutually exclusive: they likely reflect true biological variation across disease phenotypes and the influence of study design, rather than a single uniform effect of endometriosis on oocyte competence.

To resolve these inconsistencies, future clinical and translational studies should adopt standardized, rigorous designs and report key covariates explicitly. Recommended elements include: stratification by endometriosis phenotype and stage (superficial peritoneal, ovarian endometrioma, deep infiltrating) and separate analyses for ovarian-involving versus non-ovarian disease [109,110,111,112,113,114,115,116,117,118]; detailed documentation of prior surgical history (e.g., cystectomy, excision) and recent hormonal treatments or suppression protocols, with sensitivity analyses excluding recently treated patients [110,111,112,113,114,115,139]; matching or multivariable adjustment for age and ovarian reserve markers (AMH, AFC) and reporting both per-patient and per-oocyte/embryo outcomes with appropriate hierarchical (multilevel) statistical models to account for clustering; harmonization of ovarian stimulation regimens, fertilization method (ICSI vs. conventional IVF), embryo culture systems, and morphological scoring criteria across centers; standardized timing and validated assays for oxidative and inflammatory biomarkers in follicular fluid and serum, with pre-specified sampling windows relative to stimulation and retrieval [133,134,135,136,137,138,139]; adequate sample size calculations powered for clinically meaningful endpoints (live birth) and for subgroups defined by phenotype; and incorporation of molecular profiling (follicular fluid metabolomics, single-oocyte mitochondrial and spindle imaging, transcriptomics) in nested substudies to link morphological observations with mechanistic biomarkers. Explicitly stating these limitations and the above design elements in the Methods or Limitations section will clarify the interpretability of results and guide reproducible, comparable research that can determine whether morphological differences translate into clinically meaningful reductions in oocyte competence.

## 5. Modifiable Strategies for Reproductive Longevity in Endometriosis

While ovarian reserve represents a largely genetically predetermined and irreversible quantitative parameter, oocyte quality (developmental competence) is a dynamic trait that remains highly sensitive to environmental, metabolic, inflammatory, and endocrine influences. In women with endometriosis, the follicular and peritoneal microenvironments are frequently hostile, characterized by chronic inflammation, oxidative stress, altered steroid signaling, vascular dysfunction, and immune dysregulation, all of which may impair oocyte competence and reproductive outcomes.

Emerging evidence suggests that modifiable factors, including lifestyle, nutrition, environmental exposures, and individualized clinical strategies, can partially counteract these detrimental mechanisms by influencing nutrient-sensing pathways, redox balance, immune–endocrine crosstalk, epigenetic programming, and the microbiome, thereby promoting reproductive longevity despite underlying disease [231].

### 5.1. Early-Life and Anthropometric Factors: Developmental Programming of Risk

Scientific advances support the concept that prenatal and early-life exposures can reprogram reproductive health across the lifespan. Epidemiological studies indicate that both low and high birthweight are associated with a modestly increased lifetime risk of endometriosis (relative risk ≈ 1.3), consistent with a U-shaped relationship between fetal growth and disease susceptibility [232]. Evidence regarding prematurity remains inconsistent, underscoring the importance of analyses restricted to term births or adjusted for gestational age.

Body weight across the life course also appears to modulate endometriosis risk. Although early descriptions portrayed women with endometriosis as characteristically lean, it remains unclear whether low body weight is causal or a consequence of disease-related metabolic alterations. Overall, population-level data indicate an inverse association between BMI and endometriosis prevalence, with a meta-analysis reporting a 33% reduction in risk per 5 kg/m^2^ increase in BMI (RR = 0.67), albeit with substantial heterogeneity across studies [232]. Notably, some cohorts report increased risk among obese or prepubertally overweight girls, suggesting critical windows of vulnerability during development.

### 5.2. Lifestyle and Nutrition: Modulation of Nutrient-Sensing Pathways

The impact of nutrition on fertility extends far beyond caloric intake or body mass, involving evolutionarily conserved nutrient-sensing pathways that regulate cellular aging, stress resistance, and reproductive lifespan. As outlined by Templeman and Murphy (2018) [202], reproductive and somatic longevity are coordinated through three major signaling axes:

- Insulin/IGF-1 signaling, chronically hyperactivated by high-glycemic diets, accelerates ovarian aging and compromises oocyte quality.
- mTOR (mechanistic target of rapamycin) integrates amino acid and energy availability, promoting growth at the expense of autophagy and cellular repair when persistently activated.
- AMPK, activated by caloric restriction and physical exercise, enhances stress resistance, mitochondrial function, and genomic stability.

In endometriosis, an intrinsically inflammatory and estrogen-dependent disease, dietary patterns that attenuate excessive activation of insulin/IGF-1 and mTOR while promoting AMPK signaling—such as Mediterranean-style diets, low-glycemic-load regimens, and caloric restriction mimetics—may exert dual benefits by reducing systemic inflammation and slowing excessive follicular recruitment (“ovarian reserve burnout”).

Physical activity may further modulate disease activity by reducing luteal estrogen levels, increasing sex hormone–binding globulin, and dampening inflammatory signaling, although epidemiological evidence remains heterogeneous (RR ≈ 0.89 for highest vs. lowest activity) [202].

### 5.3. Dietary Components, Inflammation, and Epigenetic Modulation

Dietary composition directly influences key pathogenic mechanisms of endometriosis, including estrogen signaling, prostaglandin synthesis, oxidative stress, immune activation, and epigenetic regulation. Diets with high inflammatory potential—rich in ultra-processed foods, trans fats, saturated fats, and added sugars—have been associated with up to a fourfold increased likelihood of endometriosis, while consumption of more than two servings of red meat per day correlates with a 56% increased risk [233].

**Figure 4:**
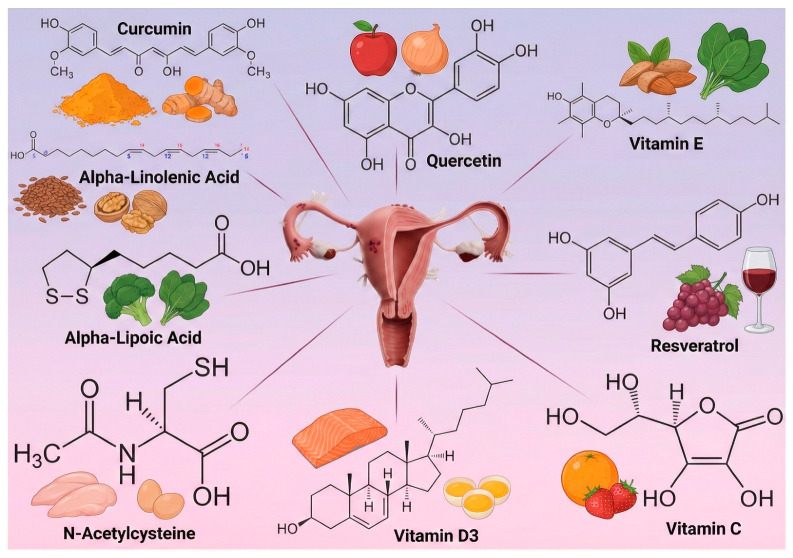
**Key dietary antioxidants and bioactive compounds modulating the reproductive microenvironment in endometriosis**. This illustration highlights the chemical structures and nutritional sources of key antioxidants, including polyphenols (Curcumin, Quercetin, Resveratrol), essential vitamins (C, E, D3), and fatty acids (ALA, Alpha-Lipoic Acid, NAC). These nutrients are essential for restoring redox homeostasis within the follicular fluid, counteracting the detrimental effects of ROS on oocyte competence, and mitigating systemic inflammatory pathways, such as prostaglandin synthesis and mTOR signaling, implicated in the pathogenesis and symptomatic progression of the disease. Adapted from [234,235,236,237,238,239,240,241,242,243,244,245,246,247,248,249,250,251].

Conversely, higher intake of antioxidants and micronutrients appears protective, as various bioactive compounds can directly modulate the biochemical environment of the reproductive system (Figure 4). Reduced concentrations of vitamins C and E have been observed in the follicular fluid of women with endometriosis, suggesting compromised antioxidant defenses. Omega-3 polyunsaturated fatty acids may exert anti-inflammatory effects by shifting prostaglandin synthesis away from PGE_2_ and PGF_2_α, although randomized supplementation trials show mixed clinical results.

Importantly, dietary factors such as folate, polyphenols, and caloric intake influence DNA methylation, a process deeply implicated in endometriosis pathogenesis. Bioaccumulated lipophilic contaminants present in animal fats may further interfere with endocrine and epigenetic regulation, although direct causal evidence remains limited [234].

### 5.4. The Microbiome–Estrobolome Axis

The human microbiome plays a fundamental role in immune maturation, metabolic regulation, and estrogen homeostasis. Diet is a major determinant of microbial composition, and dysbiosis has been implicated in multiple chronic inflammatory diseases, including endometriosis.

Women with endometriosis often exhibit altered microbial profiles across oral, vaginal, fecal, and cervical compartments, with increased abundance of potentially pathogenic taxa such as *Escherichia*, *Enterococcus*, *Tepidimonas*, and *Fusobacterium*. While not universally observed across cohorts, accumulating evidence suggests that endometrial dysbiosis may interact with local estrogen metabolism.

Recent studies support disruption of the estrobolome, the collection of microbial genes involved in estrogen metabolism. β-glucuronidase–producing bacteria can deconjugate estrogen metabolites, increasing local estrogen bioavailability and potentially promoting epithelial proliferation and lesion persistence [235]. These findings provide a mechanistic link between diet, microbiome composition, estrogen signaling, and disease activity.

Low-FODMAP diets, well established for irritable bowel syndrome (IBS), may offer substantial symptomatic benefit in women with coexisting IBS and endometriosis, with reported improvement rates up to 72%, supporting a multidisciplinary dietary approach [232].

### 5.5. Oxidative Stress, the Antioxidant Paradox and Emerging Nanotoxico-Logical Risks

Oxidative stress is a central mediator of endometriosis-associated infertility. Follicular fluid from affected women exhibits elevated lipid peroxidation markers (e.g., 8-isoprostane) and reduced total antioxidant capacity, leading to meiotic spindle instability, mitochondrial dysfunction, and impaired ATP production, all of which compromise oocyte competence [115].

However, antioxidant supplementation requires caution. Physiological ROS signaling is essential for ovulation and normal oocyte maturation. Thus, targeted, time-limited antioxidant strategies (e.g., vitamins C and E, resveratrol) aimed at restoring redox homeostasis, rather than indiscriminate chronic use, are more biologically plausible [236].

### 5.6. Environmental Exposure and Emerging Nanotoxicological Risks

Beyond classical endocrine-disrupting chemicals such as phthalates and bisphenols, emerging evidence highlights the reproductive toxicity of nanomaterials, which can cross the blood–follicle barrier. Accumulation of nanoparticles in ovarian tissue may trigger local inflammation, disrupt granulosa cell steroidogenesis, and induce premature apoptosis of cumulus cells, thereby indirectly impairing oocyte quality.

In women with endometriosis, who already exhibit baseline inflammation and barrier dysfunction, cumulative xenobiotic exposure may act as a “second hit.” Risk-reduction strategies include minimizing exposure to heated plastics, improving water filtration, and the informed selection of cosmetic and pharmaceutical products [237].

We also provide a summary of dietary patterns and nutrients that have been evaluated in endometriosis research (Table 2 and Table 3). Several dietary approaches, including the Mediterranean diet, anti-inflammatory diets, gluten-free diets, low-FODMAP diets, and plant-based diets, have been primarily investigated in relation to pain symptoms, inflammatory pathways, gastrointestinal manifestations, and quality of life. In parallel, specific nutrients and bioactive compounds—such as omega-3 fatty acids, vitamin D, antioxidants, polyphenols, and iron—have been explored for their potential roles in modulating inflammation, oxidative stress, immune responses, and symptom management [233].

### 5.7. Clinical Optimization and Personalized ART Strategies

Large retrospective analyses challenge the assumption that endometriosis uniformly impairs oocyte morphology. Data from over 6000 oocytes indicate that quantitative deficits (fewer retrieved MII oocytes), rather than qualitative morphological abnormalities, may represent the primary limitation when age is controlled [6].

This insight shifts clinical focus toward personalized ovarian stimulation strategies, including optimized gonadotropin selection (e.g., inclusion of LH activity), careful trigger timing to synchronize nuclear and cytoplasmic maturation, and individualized protocols that account for inflammatory and metabolic context.

## 6. Conclusions and Future Directions

Endometriosis exerts multifaceted effects on ovarian function and oocyte competence through intertwined inflammatory, oxidative, metabolic, and epigenetic mechanisms. Current evidence supports a model in which local lesion biology (iron overload, ROS and cytokine imbalance), altered immune surveillance, and metabolic reprogramming converge to reduce follicular yield and, in some contexts, impair oocyte and embryo quality [109,110,111,112,113,114,115,116,117,118,123,133,134,135,136,137,138,139]. However, clinical outcomes are heterogeneous and context-dependent, reflecting disease phenotype, prior treatments, patient age and ovarian reserve, as well as differences in study design and laboratory practice.

Key knowledge gaps remain. Indeed, it is not yet established which molecular perturbations consistently translate into clinically meaningful reductions in oocyte competence and live birth, which lesion phenotypes pose the greatest reproductive risk, and which patient subgroups will benefit from targeted adjunctive therapies. The relative contributions of local ovarian toxicity (endometrioma-proximal iron/ROS) versus systemic inflammatory or epigenetic effects on embryo implantation and pregnancy maintenance are incompletely defined [110,111,112,113,114,115,129,130,131,132,133,134,135,136,137,138,139].

To the best of our knowledge, we suggest methodological priorities for future research, which will include: i) Phenotype-specific cohorts: studies must stratify patients by endometriosis phenotype and stage (superficial, ovarian endometrioma, deep infiltrating) and report ovarian-involving versus non-ovarian disease separately [109,110,111,112,113,114,115,116,117,118]; ii) Rigorous covariate control: prospective designs should match or adjust for age, AMH/AFC, prior surgery, and recent hormonal treatments; report both per-patient and per-oocyte/embryo outcomes using hierarchical statistical models to account for clustering. iii) Standardization and harmonization: adopt standardized stimulation protocols, fertilization methods (ICSI vs. conventional IVF), embryo culture systems, and morphological/kinetic scoring to reduce inter-study variability. iv) Pre-specified biomarker sampling and harmonize timing and validated assays for follicular fluid and serum markers (ROS, MDA, 8-OHdG, iron, cytokines and SIRT3/SOD2) with clear windows relative to stimulation and retrieval [133,134,135,136,137,138,139].

Future work should pair clinical endpoints with mechanistic readouts in nested designs: combine follicular-fluid and granulosa-cell assays with single-oocyte assessments (spindle imaging, mitochondrial copy number and transcriptomics) and embryo phenotyping (morphokinetics, euploidy). Such integrative datasets will permit causal inference linking specific molecular insults (iron/ROS, cytokine milieu and miRNA/epigenetic signatures) to discrete outcomes (oocyte maturation, fertilization, blastulation, implantation and live birth) and identify biomarkers with prognostic or predictive value [41,42,50,93,94,95,96,123,133,134,135,136,137,138,139].

Preclinical evidence supports several actionable strategies, including iron chelation/ferroptosis modulation, antioxidant/redox modulation, NF-κB/COX-2 pathway inhibition, macrophage repolarization, and antifibrotic approaches, that warrant translation into carefully designed clinical trials [123,147,148,149]. Early-phase trials should prioritize safety and ovarian reserve preservation, include mechanistic biomarker endpoints, and target well-defined phenotypes (for example, patients with endometrioma-proximal oxidative signatures). Adaptive trial designs and biomarker-guided randomization may accelerate identification of effective adjuncts to surgical and hormonal management.

Until definitive interventional data are available, clinicians should individualize management by integrating phenotype, ovarian reserve, prior treatments, and patient priorities. Where endometriomas are present, counseling should address the likely reduction in oocyte yield and the uncertain impact on intrinsic oocyte quality; decisions about cystectomy versus conservative management should weigh surgical risks to ovarian reserve against symptomatic and reproductive goals [109,110,111,112,113,114,115,116,117,118].

Advancing reproductive outcomes in women with endometriosis requires coordinated, multidisciplinary research that links rigorous clinical phenotyping with mechanistic biomarkers and translational trials. By standardizing methods, powering studies for clinically relevant endpoints, and embedding mechanistic substudies, the field can move from descriptive associations to actionable interventions that preserve fertility and improve pregnancy success for affected women.

## Figures and Tables

**Table 2:** **Summary of dietary patterns investigated in endometriosis research and their reported effects on symptoms and underlying biological mechanisms**.

Dietary Pattern/Intervention	Outcomes Investigated	Mechanisms Explored	References
Anti-inflammatory diet	Pregnancy-related outcomes, including preterm birth and pre-eclampsia	Reduction of systemic inflammatory markers	Kyozuka et al., 2021 [238]
Gluten-free diet	Pelvic and abdominal pain	Attenuation of inflammatory processes	Marziali et al., 2012 [235], Losurdo et al., 2017 [236]
High-fiber diet	Risk of endometriosis; serum estrogen concentrations	Estrogen regulation; increased gut microbiome diversity; modulation of microbiota-related metabolic pathways	Parazzini et al., 2013 [241]
Low-FODMAP diet	Gastrointestinal symptoms and IBS-related symptoms associated with endometriosis	Restoration of gut barrier function; mast cell stabilization	Moore et al., 2017 [242]
Low-nickel diet	Chronic pelvic pain, dysmenorrhea, and dyspareunia	Modulation of inflammatory responses	Borghini et al., 2020 [243]
Mediterranean diet	Dyspareunia, dyschezia, and dysmenorrhea	Enhanced antioxidant capacity; anti-inflammatory effects	Nirgianakis et al., 2022 [244]

**Table 3:** **Overview of selected nutrients and bioactive compounds evaluated in endometriosis research, with reported clinical outcomes and proposed mechanisms of action**.

Nutrient/Compound	Outcomes Investigated	Mechanisms Explored	References
Alpha-lipoic acid (ALA)	Oxidative stress, progression of endometrial implants, and inflammatory markers	Reduction of oxidative stress; decreased TNF-α levels in serum and peritoneal fluid; inhibition of cellular adhesion and invasion	Pinar et al., 2017 [245]
Curcumin	Endometrial cell proliferation, estrogen (E2) production, and inflammatory cytokines (TNF-α, IL-6, IL-8)	Anti-inflammatory activity; reduction in E2 levels; modulation of inflammatory signaling pathways	Zhang et al., 2013 [246]
Fish oil (omega-3 PUFA)	Pain severity and size of endometriotic lesions	Anti-inflammatory effects; downregulation of prostaglandin synthesis	Nodler et al., 2020 [247]
N-acetyl-cysteine (NAC)	Pelvic pain, dysmenorrhea, dyspareunia, and ovarian endometrioma/cyst size	Reduction of cyst size; modulation of pain-related pathways	Porpora et al., 2013 [248]
Quercetin	Endometrial cell proliferation and prostaglandin and leukotriene production	Anti-inflammatory and anti-proliferative activities; inhibition of prostaglandin and leukotriene synthesis	Jamali et al., 2021 [249]
Resveratrol	Pain, inflammatory and oxidative status, and proliferative activity	Anti-inflammatory, antioxidant, and anti-proliferative effects; reduction of matrix metalloproteinase activity	Novakovic et al. [250]
Resveratrol combined with drospirenone/ethinylestradiol	Pain and dysmenorrhea	Modulation of pain-related and hormonal pathways	Maia et al., 2022 [251]
Vitamin D	Pelvic pain, C-reactive protein levels, and total antioxidant capacity	Immunomodulatory activity; inhibition of angiogenesis; anti-inflammatory effects; modulation of vitamin D–related pathways	Abolfazel et al., 2021 [252]
Vitamins C and E (combined therapy)	Dysmenorrhea, dyspareunia, and pelvic pain	Increased antioxidant capacity	Amini et al., 2021 [252]

## Data Availability

No new data were created. During the preparation of this manuscript, the authors used Copilot, public online version, for the purposes of Medline on a specific topic and table generation. The authors have reviewed and edited the output and take full responsibility for the content of this publication.

## Abbreviations

The following abbreviations are used in this manuscript:

RSA	Recurrent Spontaneous Abortion
LHR	LH Receptor
ROS	Reactive Oxygen Species
ART	Assisted Reproductive Technology
PCOS	Polycystic Ovary Syndrome
NOXs	NADPH Oxidases
OXPHOS	Oxidative Phosphorylation
PDH	Pyruvate Dehydrogenase
PPP	Pentose Phosphate Pathway
DE	Deep Endometriosis
RIF	Recurrent Implantation Failure
ZP	Zona Pellucida
GC	Granulosa Cell
EMT	Endothelial–Mesenchymal Transition
ECM	Extracellular Matrix
PI3K–AKT	Phosphoinositide 3-kinase – Protein Kinase B
AKT	Protein Kinase B
IL	Interleukin
NK	Natural Killer
PGE_2_	Prostaglandin E2
RA	Retinoic Acid
ESR	Estrogen Receptor
EMT/EndMT	Epithelial–Mesenchymal and Endothelial–Mesenchymal Transition
SASP	Senescence-Associated Secretory Phenotype
IVM	In Vitro Maturation
mtDNA	mitochondrial DNA
